# The Little Fly that Could: Wizardry and Artistry of *Drosophila* Genomics

**DOI:** 10.3390/genes5020385

**Published:** 2014-05-13

**Authors:** Radoslaw K. Ejsmont, Bassem A. Hassan

**Affiliations:** 1 VIB Center for the Biology of Disease, VIB, 3000 Leuven, Belgium; 2 Center for Human Genetics, University of Leuven School of Medicine, 3000 Leuven, Belgium

**Keywords:** *Drosophila*, genetics, genomics

## Abstract

For more than 100 years now, the fruit fly *Drosophila melanogaster* has been at the forefront of our endeavors to unlock the secrets of the genome. From the pioneering studies of chromosomes and heredity by Morgan and his colleagues, to the generation of fly models for human disease, *Drosophila* research has been at the forefront of genetics and genomics. We present a broad overview of some of the most powerful genomics tools that keep *Drosophila* research at the cutting edge of modern biomedical research.

## 1. Introduction

The Human Genome Project, on its way to producing an assembled genome of *Homo sapiens*, has gone through several test runs yielding sequenced genomes of other organisms of high relevance for research into human development and disease. The first published genome of a free-living organism was that of the proteobaterium *Haemophilus influenzae* [1], followed by sequencing of the genome of *Saccharomyces cerevisiae* yeast, the first eukaryotic genome sequenced [2], and the genome of *Caenorhabditis elegans*, the first genome of a multicellular organism and the first animal genome [3]. The second animal genome sequenced was that of the fruit fly *Drosophila melanogaster* [4]. In this review, we discuss the significance of the sequencing of the *Drosophila* genome as well as the technical advances and new research avenues that have accompanied it.

## 2. Drosophila as a Model

### 2.1. In Development

The fruit fly has been studied for over a century and the lessons learned from fly research makes it almost impossible to enumerate but a few of the most notable cases. The pioneering studies that identified genes involved in *Drosophila* embryo segmentation [5,6] and establishment of segment polarity [6] were seminal for understanding conserved developmental strategies in the animal kingdom. The discovery of homeotic genes is one of the best-known examples of genes discovered in the fruit fly, and these were found to be conserved and play analogous roles in humans [7,8,9]. *Drosophila* has played a seminal role in sensory organ development research. The discovery of the *eyeless* gene [10], a fly homolog of human and mouse PAX6 [11,12], and determination of its targets [13] shed light on vertebrate eye development and led to discovery of novel disease related genes in humans [14]. The proneural gene atonal plays a crucial role in the development of *Drosophila* photoreceptor neurons [15] and chordotonal organs [16]. Its function is conserved in mammals, where its homologs Math5 and Math1 were shown to be involved in regulating formation of retinal ganglion cells [17] and inner ear mechanosensory hair cells [18].

### 2.2. In Signaling

*Drosophila* has been extensively used for studies of signaling pathways. In Hedgehog signaling, both the Hedgehog ligand itself [6,19,20] and its receptor Patched [6,21,22] were first identified in the fly, though the link between the two was first established in mammals [23,24]. The ligand of the Wnt signaling pathway turned out to be a well-known *Drosophila* segment polarity protein, *i.e*., Wingless. The Wnt receptor, Frizzled [25], and several other signal transduction cascade members were identified in the fly as members of the Wnt pathway [26,27,28]. The planar cell polarity (PCP) pathway is yet another example of a signaling cascade in which key players and mechanisms of action have been, to a large extend, identified in *Drosophila* [29,30]. The Notch signaling pathway, associated with cell fate control, lateral inhibition, and signal integration during development, has been discovered and extensively studied in fruit flies [31,32,33]. Finally, major components and mechanisms of action of the Hippo signaling pathway have been described in *Drosophila* [34,35,36]. All these pathways play major roles in human development and disease.

### 2.3. In Disease

Over the past two decades the fruit fly became an increasingly popular model organism for the study of human disease, with focus on neurodegenerative [37] and neuromuscular [38] diseases as well as cancer [39]. Neurological diseases that have been modeled in *Drosophila* include trinucleotide repeat disorders [40,41,42], Alzheimer’s disease [43,44,45,46], Parkinson’s disease [47,48], amyotrophic lateral sclerosis [49,50], and dystrophy [51]. Other examples that include use of the fruit fly model are studies of alcohol abuse [52,53], cocaine addiction [54], obesity [55] and diabetes [56], cardiac diseases [57], and asthma [58]. *Drosophila* has been demonstrated to be a great model to identify tumor suppressor genes [59] or genes involved in metastasis [60]. Thanks to the conservation of major signaling pathways, tumor suppressors and oncogenes, various fly cancer models have been established. Understanding how signal transduction pathways like Hippo, Notch, Dpp or JAK-STAT affect tumor formation was aided by research in fruit flies [61,62,63]. *Drosophila* has been used as a model for tumor invasion and metastasis [64], and as a platform to identify novel therapeutic targets [65].

## 3. Meet the Drosophila Genome

The *Drosophila* genome is estimated to be approximately 200 Mb, with one third of it forming pericentric heterochromatin [66]. It is organized on three autosomes (numbered 2, 3 and 4) and sex chromosomes, X (also referred to as the first chromosome) and Y. The initial assembly of the fruit fly genome was published in March 2000, after almost a year of whole genome shotgun sequencing. The first published assembly, referred to as Release 1 of the genome, included 13,991 genes encoding for 14,080 peptides. Over two thirds of annotated genes were assigned gene ontology (GO) terms upon annotation. The initial assembly contained ~1300 gaps in mapped sequences [4] that were filled with subsequent releases.

The third release of the genome was the first that included pericentric heterochromatin sequences [67]. The mutations indicated in the sequenced strain’s genotype, as well as several other identified mutations, have been corrected with wild-type sequence [68]. With that release, a comprehensive set of resources were published, including a library of full-length cDNAs for 40% of genes [69] and an atlas of gene expression patterns during embryogenesis [70]. Sequence analysis provided insights into transposable elements within the genome [71], core promoter structures [72], and largely improved annotation of gene models [68].

The current, fifth assembly of the genome has closed all but 9 gaps in the main assembly. The sequenced genome covers over 120 Mb of euchromatin, and over 9 Mb of mapped and over 10 Mb of unmapped heterochromatin. The current annotation revision contains 13,942 protein coding genes and over 2354 non-coding RNA genes, including ribosomal (rRNAs), transport (tRNAs), micro- (miRNAs), and small nuclear (snRNA) and small nucleolar (snoRNA) RNAs [73]. Through genome analysis, fruit flies have been found to contain complex gene structures. Approximately 7.5% of all genes, including non-coding RNAs, are located within the introns of other genes. Messenger RNAs for about 15% of genes overlap with mRNAs of genes on the opposite strands. Over 30 genes have been identified as dicistronic, *i.e.*, producing single mRNA encoding for two separate protein products through independent translation initiation events [68]. Over 30% of *Drosophila melanogaster* genes were found to be alternatively spliced [74], yielding a diverse set of almost 30,000 protein-coding transcripts [73]. The next release of the genome assembly (Release 6) is expected this year (2014).

Improved assembly and annotation of the fruit fly genome was possible not only due to new sequencing data, but also thanks to advances in bioinformatics tools. An integrated computational pipeline and a tailored database schema have been developed to facilitate genomic data storage and automated sequence annotation [75]. Computed annotations have been manually curated by experts and to aid in this task, a dedicated annotation editor was developed [76]. Finally, automated genome annotation in general requires the use of computational tools, some of which were first applied in the *Drosophila* genome project [72,77].

## 4. Genomes by the Dozen

Analysis of coding parts of the genome can be facilitated by comparison of genomic sequences with sequences of cDNAs originating from the same species. Most of the DNA in the majority of species, however, is non-coding. One approach to identify functional non-coding DNA segments, such as *cis*-regulatory elements, relies on finding conserved regions or motifs across related species. This naturally requires having more than one genome sequenced and was a driving force behind sequencing of the genomes of *Schizosaccharomyces* *pombe* [78] and *Caenorhabditis briggsae* [79] in the yeast and worm research communities, respectively. In the *Drosophila* genus, the comparative genomics era began with sequencing of the *Drosophila pseudoobscura* genome [80]. The two genomes were found to be very similar, despite 25–55 million years of evolutionary divergence. Synteny is preserved in blocks containing 10.7 genes on average, which corresponds to ~83 kb. The vast majority of synteny breaks were caused by intrachromosomal rearrangements. On average, ~48% of the base pairs are conserved between these two species.

The next advance in *Drosophila* comparative genomics came with sequencing of ten further species, *Drosophila* *sechellia*, *simulans*, *yakuba*, *erecta*, *ananassae*, *persimilis*, *willistoni*, *mojavensis*, *virilis*, and *grimshawi*. These species span a broad spectrum of morphologies, ecologies, and behaviors, yet have identical body plans and very similar life cycles [81]. Furthermore, these species share approximately 70% of their genes. Genome sizes estimated by flow cytometry vary between 130 Mb in *D. mojavensis* to 364 Mb in *D. virilis* [66]. The synteny conservation between sequenced species varies with an average of 122 genes per block between *D*. *melanogaster* and *simulans* down to 8 genes per block between *D*. *melanogaster* and *grimshawi*. Overall genome size, number of genes, distribution of transposable element classes, and patterns of codon usage are all very similar across the 12 sequenced genomes. At a finer scale, however, the number of structural changes and rearrangements is larger, including rearrangements of genes within the Hox cluster or highly dynamic sizes and content of multigene families [81].

Together, the 12 *Drosophila* genomes provide a solid platform for annotation and analysis of both coding and non-coding DNA. This unprecedented dataset enabled the use of evolutionary signatures—specific patterns of change in DNA elements upon selection—for *de novo* prediction and correction of previously annotated protein-coding gene models [82], non-coding RNAs, and transcription factor (TF) binding sites [83]. Identification of TF binding motifs has traditionally been based on DNA alignments. Alignment-based methods can also be used for the identification of *cis*-regulatory modules (CRMs), which are comprised of a number of TF binding motifs [84]. In many cases, however, the number and order of individual motifs varies between species, especially when these are distant, while preserving regulatory outcome. To address such cases, alignment-free approaches have also been developed [85,86].

## 5. Genomes by Population

*Drosophila* provides an unmatched set of resources for studying quantitative traits [87]. In the post genomic era, genome-wide association studies (GWAS) have become a preferred method for analyzing complex traits. The GWAS methodology is now routinely and successfully applied in the identification of human disease-associated genes [88]. Two fruit fly resources, the *Drosophila* Genetic Reference Panel (DGRP) [89] and the *Drosophila* Synthetic Population Resource (DSPR) [90], offer large sets of sequenced and mapped fly lines tailored for GWAS and quantitative trait loci (QTL) mapping.

The DGRP is a collection of more than 200 fully sequenced recombinant inbred lines (RILs) that were established from mated females collected from a market in Raleigh, North Carolina, USA. The genomic sequences of these lines contain over 4.5 million single nucleotide polymorphisms (SNPs), over one hundred thousand polymorphic microsatellites, and over 36 thousand transposable elements [89]. The DGRP has been extensively characterized and in addition to detailed genomic sequence analysis includes microarray [91] and RNA-seq [92] datasets for selected lines. To date, numerous genome-wide association studies have been published on various traits using DGRP, including oxidative stress [93], mitochondrial function [94], viral infection resistance [95], and sleep [96].

The *Drosophila* Synthetic Population Resource uses a different approach. Over 1,700 DSPR RILs were established from 15 isogenic founder lines created from geographically distinct *Drosophila* populations. The founder lines were split in two groups of eight (with one line in both groups) and mixed for 50 generations to create two synthetic populations, from which two sets of RILs were established. The founder lines were fully sequenced and each RIL was mapped using restriction-site associated DNA (RAD) markers onto the founders’ sequence with 17 kb median resolution. The number of SNPs in the founder lines exceeds 1.6 million [90]. The DSPR is complementary to DGRP and both resources can be used together for cross-validation and to increase the mapping power [97].

## 6. Decoding the Genome’s Secrets

### 6.1. The modENCODE Project

The information encoded by genomes goes far beyond a simple trinucleotide code used to translate nucleic acid sequence into protein. A plethora of information is hidden within introns, UTRs, non-coding RNAs, *cis*-regulatory elements, and chromatin marks. These elements are known to regulate where and when a gene product is expressed. The human ENCODE (ENCyclopedia Of DNA Elements) project [98] aims to identify and understand the information carried by the human genome. The modENCODE project is the model organism counterpart of ENCODE with focus on two species *C*. *elegans* [99] and *D*. *melanogaster* [100]. The fruit fly modENCODE data includes high-throughput transcriptome sequencing (RNA-seq), chromatin immunoprecipitation followed by sequencing (ChIP–seq) for transcription factor binding sites and histone modifications, DNA replication patterns, and nucleosome occupancy. The samples have been collected from 12–30 developmental time points of the sequenced *D. melanogaster* strain and from several cell lines [100].

### 6.2. The Transcriptome

Comprehensive transcriptomics data has redefined gene models for 75% of fly genes by adding new exons or splice variants. The majority of annotation changes were supported by direct cDNA evidence. Analysis of transcription start sites (TSSs) for over half of *Drosophila* genes resulted in identification of over 1500 novel promoters. The structural analysis of RNA-seq-identified transcripts that did not seem to encode proteins revealed that a majority of them has no thermodynamically stable secondary structure, suggesting structure-independent functions. Among structural non-coding RNAs, several hundred novel small regulatory RNAs (miRNAs, siRNAs, and piRNA) have been identified. Additionally, transcription start sites for both protein coding and non-coding RNAs have been derived from the presence of chromatin marks characteristic of transcriptionally active regions, such as H3K4me3 enrichment, H3K9ac, and presence of RNA polymerase II in TSS-proximal regions [100].

### 6.3. Chromatin Landscape

Eukaryotic genomes are organized into large domains that exhibit distinct chromatin properties [100]. Analysis of large-scale organization of the chromatin landscape has revealed unexpected complexity and plasticity among different cell types. Some regions in the usually silent pericentric heterochromatin exhibited surprisingly high gene expression activity. Conversely, large regions of normally transcriptionally active euchromatin harbored histone marks (H3K9me2) typical for heterochromatin [100,101]. Chromatin signatures characteristic of various functional elements have been identified by ChIP-chip for 18 histone modifications (both activating, such as H3K4me, H3K9/18/27ac, H2B ubiquitination and repressive, such as H3K9me2/3 or Polycomb associated H3K27me3) and variants (H1, H4) from several cell lines and developmental stages. Correlating chromatin signatures with transcriptome and protein binding data (replication factors, insulator-binding proteins, and transcription factors) helped identify marks specific for promoters, actively transcribed regions, introns, insulators, and origins of replication [100]. The presence of specific chromatin marks was found to correlate with the physical properties of chromatin, where transcriptionally active chromatin exhibited high solubility and high nucleosome-turnover rates [100]. Computational analysis of combinatorial patterns of histone modifications revealed distinct chromatin states associated with active TSSs, exons, introns, and other open chromatin as well as closed chromatin states [100,102,103].

### 6.4. Transcriptional Regulation

The modENCODE project has identified binding sites for almost 40 transcription factors through both ChIP-chip and ChIP-seq. The analysis has revealed that out of nearly 40,000 identified unique binding sites found, 5% are bound by 8 different transcription factors or more and are considered High Occupancy Target (HOT) regions. Furthermore, almost 40% of the sites can be bound by more than two factors [103]. The HOT regions exhibit decreased nucleosome density, increased nucleosome turnover and often colocalize with TSS and ORC (origin recognition complex) binding sites, suggesting interplay between chromatin regulation, TF binding, and DNA replication [100,103]. In total, modENCODE ChIP experiments revealed over 500 silencers, 2300 new promoters, over 14 candidate CBP-bound *cis*-regulatory elements, and over 7500 putative insulators [103]. Pairwise analysis of binding site co-occurrence has revealed over 800 known and putative transcription factor co-binding interactions. Binding sites for transcription factors regulating biologically opposing roles exhibited negative associations. The modENCODE TF binding data sets combined with external data were used to construct a network covering over 80 transcription factors and characterizing over 800, largely novel, regulatory interactions. Binding site co-occurrence among various analyzed promoters corresponded to temporal co-expression of the respective target genes, supporting the existence of combinatorial transcription factor codes [103].

## 7. The Fruit Fly Toolkit

### 7.1. Getting Constructs in

*Drosophila* is famous for its extensive range of forward and reverse genetics tools. The powerful toolkit was primed by the discovery of P transposable element-based germline transformation [104]. This revolutionary development allowed, for the first time, efficient delivery of foreign DNA into the genome. Development of the vast majority of *Drosophila* tools required at some stage use of P-element or other transposon systems. P-elements were used for gene cloning [105], genetic rescue [106], and as potent mutagens by their insertion [107] or excision [108]. P-element insertions have enabled the creation of enhancer traps, thus allowing visualization of gene expression patterns using genetically encoded reporters [109]. The *Drosophila* Gene Disruption Project used P-elements to create single transposon insertions in over 30% of fly genes [110]. The remaining genes, due to target sequence bias of P-elements, are currently targeted using other transposons [111]. The catalog of transposons that can be used for fly transformation has been expanded over the years and includes mariner [112,113], Minos [114,115], and piggyBac [116,117]. Each of these transposable elements, except for Minos that seems to insert randomly, has its hot and cold spots, but failure to target a certain region can often be addressed by using a different transposon [111].

While random, transposon-mediated transgenesis is desirable for gene disruption or genomic targeting, but integration of reporter or rescue constructs calls for more control over the locus where these integrate, thus reducing the chance of position effects that can strongly influence gene expression [118,119]. Early attempts to repeatedly target a specific locus in the fruit fly genome were based on transposon homing [120]. Short regulatory sequences from *Polycomb* target genes or from the *linotte* locus included in the transposon were shown to increase the likelihood of such transposon landing in the vicinity of genomic regions bearing these sequences [120,121]. The low resolution (30 bp) and efficiency (20% of insertions) of this homing technique prompted further developments in the field. The introduction of an irreversible, site-specific recombinase from the phiC31 phage ushered in a new era in fly transgenesis. The phiC31 integrase catalyzes unidirectional recombination between two attachment sites, attP and attB, leading to the formation of attL and attR sites [122]. A circular construct harboring an attB site can be efficiently and specifically integrated into an attP site located on the genome [123]. The phiC31 integrase system has, for the first time, enabled transformation of flies with BAC-sized constructs [124]. The integrase can be expressed from mRNA co-injected with the construct [123] or from the genome under a germ-line specific promoter, the latter method being more efficient [125]. Several dozens of attP landing lines have been created and tested [123,124,125,126], creating unlimited possibilities to combine transgenes.

### 7.2. Express What You Want, Where You Want, and When You Want

P-element transgenesis has enabled the creation of a plethora of other *Drosophila* tools, of which the Gal4/UAS system is the most notable example. The system is based on yeast transcription factor gene *GAL4* fused to a minimal promoter. This construct is randomly inserted into the genome, for instance by means of P-element transgenesis, hitchhiking nearby enhancers and creating an enhancer trap. Alternatively, enhancer sequences can be cloned upstream of the minimal promoter and other arrangements, with enhancers cloned elsewhere, for example within introns, are also possible. The second component of the system is the Gal4 binding site, known as the upstream activation sequence (UAS), driving expression of the target gene. The combination of a *GAL4* enhancer trap with a UAS-driven target enables expression of the gene of interest in the desired tissues or cell types [127]. Several collections of *GAL4* enhancer traps have been created using P-element [128,129] and piggyBac [110,130] insertions. The enhancer trap resources have recently been supplemented by large collections of cloned enhancers driving expression of Gal4 [131,132]. The Gal4 expression pattern can be refined spatially [133] or temporarily [134] using the Gal80 repressor [135]. Further control over Gal4-driven expression can be obtained using variants requiring drugs for activation [136,137,138,139]. Today, the Gal4-UAS system is one of several binary expression systems available in *Drosophila*. Other examples include the LexA transactivator that binds LexOp sites [140] and the Q system with QF transactivator, QUAS binding sites and the QS repressor whose activity can additionally be drug controlled [141]. The existing binary systems can be combined to provide fine control over target expression pattern or for simultaneous targeting of different cellular populations [142].

### 7.3. Mutant Tissue on Demand

**Figure 1:**
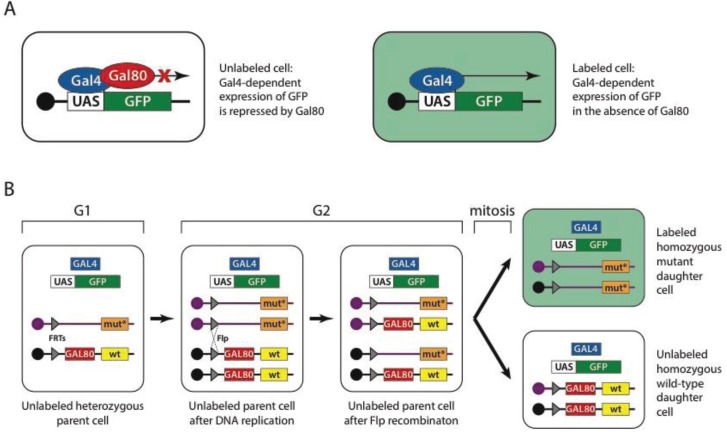
Mosaic analysis with a repressible cell marker (MARCM). (**A**) Gal4 transcription factor (blue oval) drives expression of green fluorescent protein (GFP) gene (green box) by binding the upstream activation sequence (UAS) (white box). This expression is repressed when Gal80 (red oval) is present. As a consequence cells that do not carry a gene encoding Gal80 but carry genes encoding Gal4 and UAS*-*GFP are marked green. (**B**) In MARCM, the *GAL80* repressor gene (red box) is carried on a chromosome that bears the wild-type allele of a gene (yellow box) of interest and a flippase recognition target (FRT) site (grey triangle) placed pericentricaly. The homologous chromosome carries a FRT site in exactly the same position and a mutant allele (orange box), but does not carry the *GAL80* gene. Cells also carry the *GAL4* gene and UAS-GFP on the other chromosomes. During G2 phase (after DNA replication), flippase mediates recombination between two FRT sites of homologous chromosomes, thus generating sister chromatids; one of which carries the wild-type allele and *GAL80* repressor and the other the mutant allele. During mitosis, sister chromatids are distributed to daughter cells, generating cells that are homozygous wild-type or homozygous mutant. Cells that are homozygous mutant are the only cells lacking the *GAL80* gene and thus are labeled with GFP. Reproduced with permission from MacMillan: Nature Protocols ©2007 [181].

The Gal4-UAS system is an important component of yet another powerful fruit fly tool, the mosaic analysis with a repressible cell marker or MARCM. Induction of mosaicism in *Drosophila* is used either for studying an otherwise lethal phenotype within a tissue of interest [143] or for marking a clone of cells within a tissue of interest [144]. Mosaics can be created using flippase (Flp) mediated mitotic recombination between homologous chromosomes [145]. In this technique, homologous chromosomes carry an insertion (usually P-element mediated) of a flippase recognition target (FRT) site. One of the chromosomes carries a wild type and the other a mutant allele of the gene of interest. In the presence of flippase, recombination events between homologous chromosomes can occur during cell division, leading to the generation of homozygous mutant cells from heterozygous precursors. The MARCM technique (Figure 1) enhances Flp-mediated mitotic recombination by uniquely labeling mutant cells using a genetically encoded marker. The mutant clone is marked with a UAS-GFP (green fluorescent protein) construct, driven by ubiquitously expressed *GAL4*. These two transgenes are usually inserted together on any chromosome, except the wild type chromosome that carries the Gal4 repressor—*GAL80* under the control of a ubiquitous promoter. The presence of the Gal4 repressor on the wild-type chromosome prevents GFP expression in both heterozygous and homozygous wild type cells [135]. The MARCM technique was later extended to label wild type cells as well [146].

MARCM, among other mitotic-recombination-based approaches, has enabled the creation of tissue specific mutant cells for genes where a mutant exists. This, however, is not yet [111,147] the case for all fruit fly genes. Post-transcriptional gene silencing by double-stranded RNA (dsRNA) [148], commonly known as RNA interference (RNAi), allows the silencing of virtually any transcript encoded by the genome [149]. *Drosophila* is not only one of the first organisms where RNAi has been used to silence genes [150,151], but it has also played an important role in studying the mechanism of dsRNA dependent gene silencing [152]. Injections of dsRNA into the *Drosophila* embryos were used to pioneer RNAi in the fruit fly. However, this mode of delivery has limited use for studying gene function in the late stages of development or in a tissue specific manner. A combination of genetically encoded hairpin-loop RNAs with a Gal4/UAS system has been introduced to address these issues and place *Drosophila* RNAi under spatio-temporal control [153]. Efficient transformation techniques developed for *Drosophila* Schneider (S2) cells [154] combined with *Drosophila* dsRNA libraries allowed RNAi screens in cell culture on a genome-wide scale [155,156,157,158]. With genome-wide libraries of fly lines carrying UAS-driven hairpin RNAs, tissue-specific RNAi screens in the whole animals became possible [159,160]. While the first library used P-element for transgenesis, thus leading to variability of hairpin RNA expression levels in different lines, the next generation of libraries followed, using phiC31-mediated insertions into a defined locus [161,162]. RNAi in flies has proven very effective and allowed for a number of large scale screens to be performed, including ones targeting muscle development [162], heart function [163], obesity [164], pain [165], glial function [166], or piRNA pathways [167]. The off-target effect, a well-known pitfall of RNA interference, has been addressed in flies by specificity control using either cross-species rescue [168,169] or engineered RNAi-refractory transgenes [170].

### 7.4. Bright Rescue

Modern classical and reverse-genetic approaches often call for reliable sources of transgenes, both to induce new and rescue induced phenotypes. Classically, clones from cDNA libraries [69] combined with the Gal4/UAS system [127] have been used to specifically express a gene of interest in target tissue. These constructs could be used either to ectopically express a gene of interest [127], rescue a mutant phenotype [171], or by using a fusion of cDNA with a fluorescent protein coding sequence to visualize the localization of a protein of interest [172]. These approaches, however, do not allow simultaneous modification, such as introduction of point mutations, truncation, tagging, and expression of a protein of interest under native or nearly native control. This usually requires a larger genomic context.

**Figure 2:**
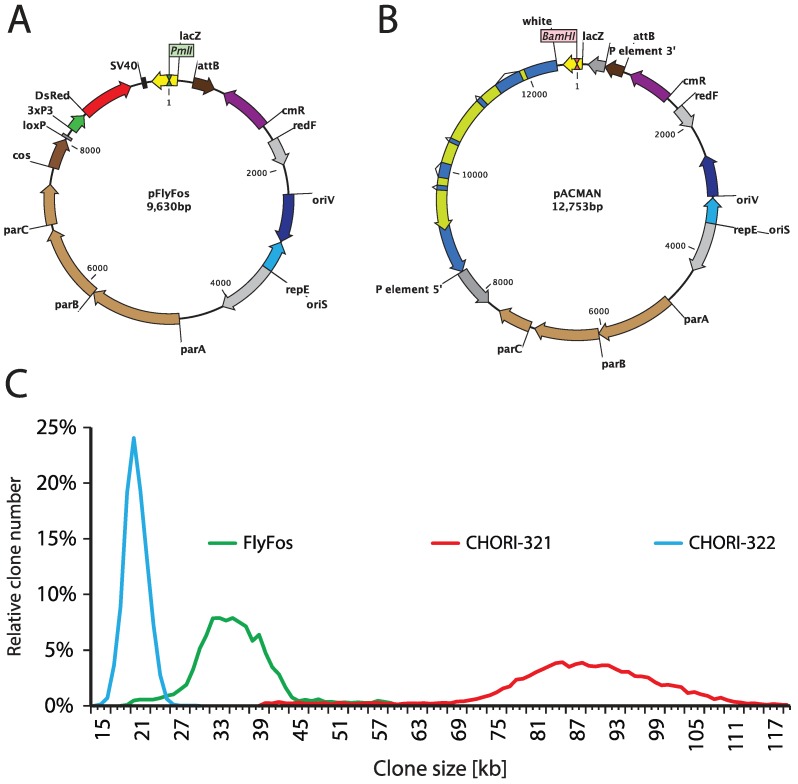
FlyFos and p[ACMAN] genomic libraries. (**A**) FlyFos library is cloned in a fosmid vector, pFlyFos. Genomic inserts were cloned into the *Pml*I site. pFlyFos features an inducible origin of replication (oriS for single copy and oriV for arabinose-inducible moderate copy maintenance), attB site for fly transgenesis, and 3xP3-dsRed as a fly-selectable marker. (**B**) p[ACMAN] libraries are cloned into the *Bam*HI site of a p[ACMAN] bacterial artificial chromosome (BAC) vector. This vector also features inducible oriS/oriV and attB site, but uses white as fly selectable marker. In addition to phiC31-mediated transgenesis, p[ACMAN] vector carrying small inserts can theoretically be used for P-element transformation. (**C**) Size distribution of FlyFos and p[ACMAN] *D. melanogaster* libraries. There are two p[ACMAN] libraries: CHORI-321 with average clone size of 83.3 kb and CHORI-322 with average clone size of 21 kb. The FlyFos library has an average clone size of 36 kb.

**Figure 3:**
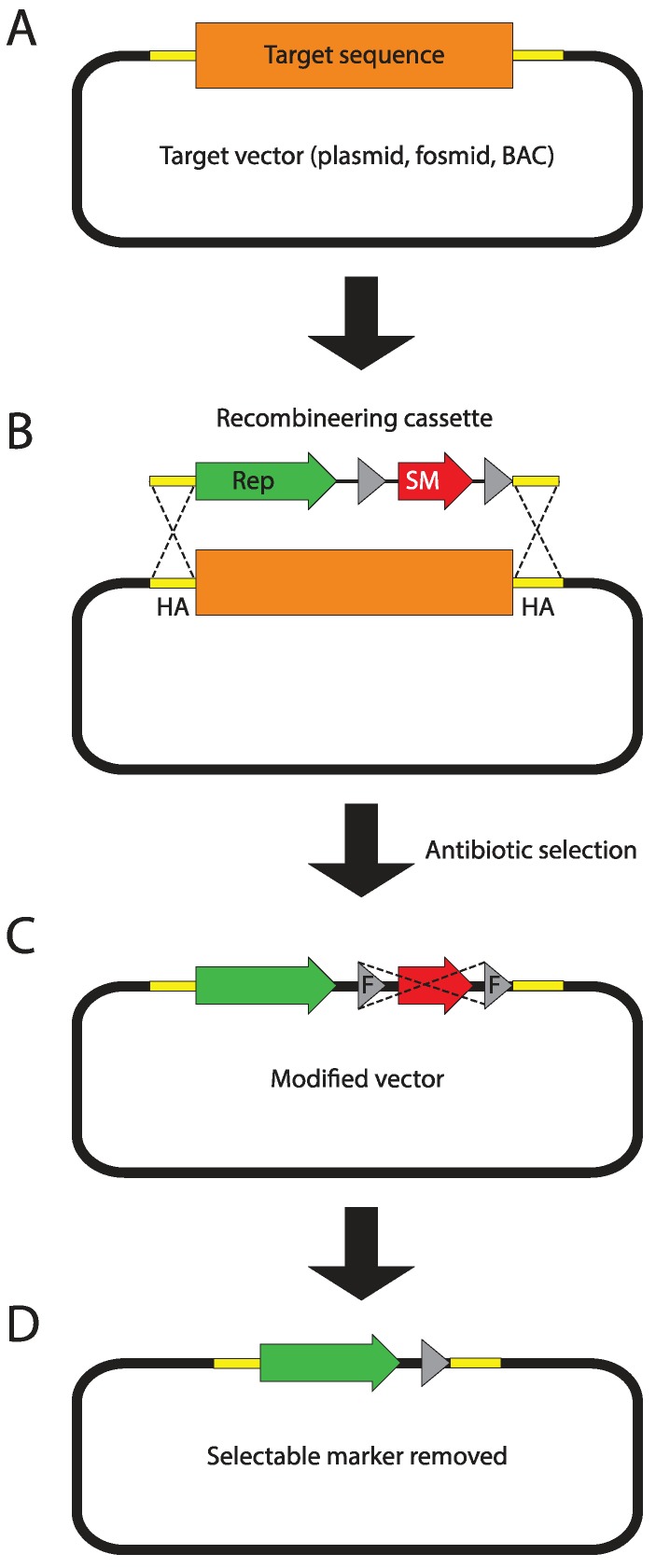
Recombineering principles. (**A**) The target sequence (orange box) is carried on a single copy fosmid or bacterial artificial chromosome (BAC) vector. The 50 bp fragments flanking the target sequence, called homology arms (HA), are depicted as yellow boxes. In this example, the target sequence will be replaced with the recombineering cassette. However, when homology arms are designed to directly follow each other, the cassette can be simply inserted into the target vector. (**B**) The PCR-amplified recombineering cassette harboring homology arms (introduced as primer overhangs) on its termini is electroporated into bacteria carrying the target vector. In the depicted example, the cassette contains a reporter (green arrow) and a flippase recognition target (FRT)-flanked (grey triangles) bacterial selectable marker (red arrow). Homologous recombinase, transiently expressed in bacteria mediates recombination between homology arms replacing the target sequence with the recombineering cassette. (**C**) Recombinant bacterial cells are selected using the selectable marker encoded in the recombineering cassette. If the selectable marker is flanked by FRT sites, it can now be removed (flipped-out) through transient expression of flippase. (**D**) The final recombineering product contains the desired sequence and a 34 bp FRT scar flanked by the homology arms.

Genome-wide libraries of fruit fly genomic DNA cloned in bacterial artificial chromosomes (BACs) or fosmids, spanning between 20 and over 100 kb, have been constructed for the purpose of genome sequencing [4]. The p[ACMAN] system (Figure 2B,C) has enabled turning them into reliable sources of modifiable genomic inserts, tailored for fly transgenesis. The centerpiece of the system is a single copy vector harboring a second, inducible medium copy origin of replication (oriV), a fly selectable marker (*white*), and attachment site (attB) for phiC31-mediated transgenesis [124]. Site-specific-recombinase-based transformation enables the insertion of constructs over 100 kb in size. Genomic inserts are subcloned into the backbone using Red/ET homologous recombination (Figure 3), also known as recombineering [173,174,175,176]. The ability to arbitrarily modify and transform large genomic constructs has fostered the development of transformation ready genomic libraries of *Drosophila melanogaster* and other fly species. Two such resources have been created so far, the p[ACMAN] [177] and FlyFos [178]. The p[ACMAN] features BAC libraries with average insert sizes of 21 and 83 kb. The vector used is similar to the one in the p[ACMAN] subcloning kit. The FlyFos system (Figure 2A,C) features 36 kb fosmid libraries for *Drosophila melanogaster* and *pseudoobscura* [169,178]. The library vector also includes an inducible oriV, attB site, and a dominant fluorescent marker, selectable in diverse insect species [179]. The liquid culture recombineering pipeline [180] introduced in the system enables high-throughput gene tagging with a variety of tags in 96-well format [178].

**Figure 4:**
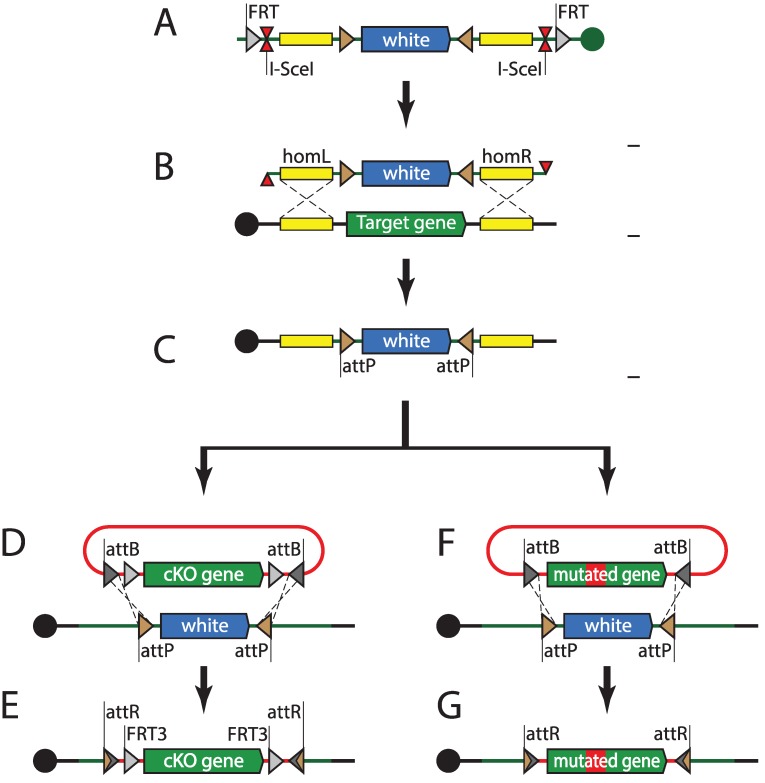
Integrase-mediated approach for gene knock-out (IMAGO). (**A**) A targeting construct harboring *white* gene flanked by attP sites, 1 kb–5 kb homology arms, I-*Sce*I meganuclease site, and flippase recognition target (FRT) sites is inserted into the fly genome using transposition or site-specific integration. (**B**) The targeting cassette is mobilized into the circular episome by flippase and subsequently linearized by meganuclease. This linear fragment induces cellular double strand break repair mechanisms and (with certain frequency) replaces the genomic locus flanked by homology arms. (**C**) Recombinant progeny are selected for *white* dominant marker. The attP sites flanking the *white* gene can be used for recombinase-mediated cassette exchange. (**D**, **F**) A plasmid containing the attB-flanked construct (cKO or a mutant allele) is injected into phiC31-expressing fly embryos and exchanges the attP-flanked *white* gene. (**E**, **G**) Recombinant progeny carrying modified alleles are selected for loss of the *white* dominant marker.

BAC and fosmid-based recombineering has enabled the introduction of modified “third alleles” of genes of interest. The powerful fruit fly genetics toolkit also allows for modifications of genes *in situ*, in their native loci. The first *in situ* genomic targeting in *Drosophila* was performed using the ends-in technique [182]. Ends-in genomic targeting relies on double strand break (DSB) repair through homologous recombination. The targeting construct contains homology arms, one of which is antiparallel to the genomic sequence, and leads to duplication of the targeted locus upon recombination. Initial targeting attempts involving linear DNA injection into the germline were unsuccessful. Inserting the targeting construct into a random locus first, via P-element transgenesis, has solved the issue. FRT sites present on the flanks of the construct were used to mobilize the targeting construct from the genome before generating DSB using I-*Sce*I nuclease [182]. Ends-out targeting uses very similar basic logic, but relies on homology arms that are both parallel to the genomic locus, therefore leading to a clean insertion or replacement [183]. Both ends-in and ends-out have provided reliable means to target genomic loci; however, at a cost of relatively low efficiency. This has made targeting the same locus with different cassettes a labor-intensive task. The integrase-mediated approach for gene knock-out (IMAGO) technique (Figure 4) combines ends-out targeting with phiC31-mediated recombinase-mediated cassette exchange (RMCE) [184]. IMAGO uses ends-out to replace the targeted locus with an attP-flanked selectable marker, which can subsequently be replaced with any desirable construct, thus enabling *in situ* gene tagging, conditional knock-outs, or functional analysis of orthologs. An alternative strategy uses a single attP site and a loxP-flanked selectable marker as the knock-out cassette [185]. Rescue constructs can then be integrated into the target locus using phiC31-mediated transgenesis, just like into any other landing site.

Genomic targeting techniques using DSBs induced in the targeting construct have proven to be robust tools. However, these approaches have a quite high price tag, because of their low efficiency. Homologous recombination with genomic loci is known to be much more effective if DSBs are introduced in the chromosome [186]. Induction of chromosomal DSBs in specific genomic loci requires designer nucleases that can target a sequence of choice. Currently three custom nuclease systems are in broad use: zinc finger nucleases (ZFNs) [187], transcription activator-like effector nucleases (TALENs), and the bacterial clustered regularly interspaced short palindromic repeat (CRISPR) system and its RNA-driven Cas9 nuclease. Double strand breaks are repaired using one of two cellular mechanisms: non-homologous end joining (NHEJ) and homologous recombination (HR). NHEJ involves processing and ligation of broken strands and usually leads to insertions and deletions [188]. However; it has also been shown to mediate efficient knock-ins in zebrafish [189]. HR requires a sequence homologous to the locus in which DSB has occurred, either from a sister chromatid, paralogous locus; or provided linear or plasmid DNA, and can, therefore, be exploited to insert or replace a genomic sequence with custom constructs [190].

**Figure 5:**
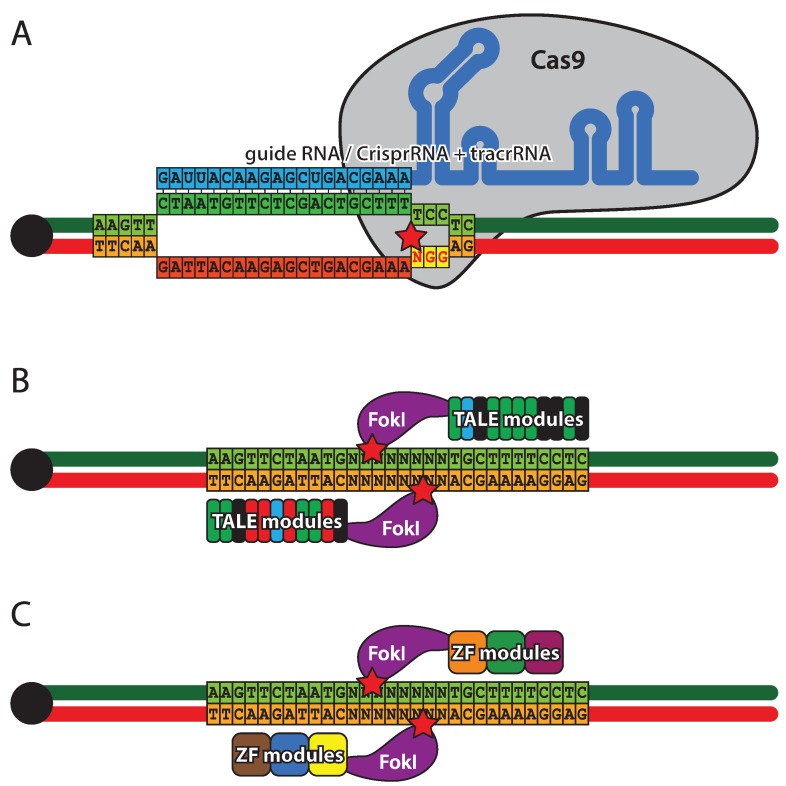
Designer nucleases. (**A**) Zink finger nucleases (ZFNs) combine a zinc finger DNA binding domain with a FokI nickase. Each zinc finger recognizes a triplet of bases and usually three to six zinc fingers are present in the targeting domain. Cleavage occurs outside the target sequence and requires a pair of ZFNs, each binding one DNA strand. (**B**) Transcription activator-like effector nucleases (TALEN), similarly to ZFNs have two domains: a DNA binding domain and a FokI nickase domain. The targeting domain is composed of 33–35 amino acid repeats, each binding a single nucleotide. The cleavage mechanism of TALENs is identical to ZFNs. (**C**) Clustered regularly interspaced short palindromic repeats (CRISPR) is a RNA driven double-stranded DNA endonuclease system. Cleavage specificity is provided by crRNA (cyan) that hybridizes with the target sequence (green). Cleavage is performed by the Cas9 protein that, in addition to crRNA, requires tracrRNA for activity. The cleavage site (star) is located between the target sequence and NGG protospacer adjacent motif, complimentary to the sequence immediately downstream of the target. crRNA and tracrRNA can be fused to form guide RNA of similar activity.

ZFNs were the first designer nuclease system (Figure 5A) to be introduced in flies [188]. They are comprised of three to four zinc-finger DNA binding modules, each recognizing three base pairs, and a FokI endonuclease. Since FokI needs to dimerize for activity, a pair of ZFNs is required for DNA cleavage [187]. The specificity and affinity of zinc-finger modules is context dependent, therefore, several strategies have been developed to achieve assembly of optimal DNA binding domains [191,192,193,194]. TALENs (Figure 5B), similar to ZFNs, are hybrids of DNA binding domains derived from transcription factors and FokI endonuclease and, as a consequence, two TALENs are required to form a functional nuclease [195,196,197]. The TALE (transcription activator-like effector) domains contain a tandem array of 15.5–19.5 repeats, each made of 34 residues, two of which provide DNA-binding specificity against a single nucleotide [198]. Due to the highly repetitive coding sequence of the TALE domain, special approaches have been developed for its efficient assembly using type IIs endonucleases [197]. CRISPR (Figure 5C), a defensive nuclease system from *Streptococcus pyogenes*, takes a completely different approach to DNA cleavage. The specificity is provided by a crRNA pairing with a 20 nt complimentary sequence within the DNA target. The cleavage is performed by Cas9 nuclease that requires trans-activating CRISPR RNA (tracrRNA) in addition to crRNA for activity. The complimentary region of the DNA target must be followed by a 3 bp PAM (protospacer adjacent motif) [199,200]. A pair of crRNA and tracrRNA can be replaced by a single hybrid guide RNA (sgRNA), thus reducing the system to two components [201]. To date, several implementations of the CRISPR system have been created in *Drosophila* [202,203,204], including transgenic flies with genomically encoded sources of Cas9 [205,206,207] and tracrRNA/sgRNA [208]. The CRISPR system has been combined with classical ends-out targeting and site-specific integrase approaches, resulting in a versatile toolkit for genome engineering [209]. It should be stated that at this stage the efficiency and specificity of all designer-nuclease-based approaches *in vivo* remains to be fully established, although CRISPR is showing great promise.

## 8. Conclusions

*Drosophila* occupies a paramount position among model organisms, largely due to the variety of genetic tools unique to the fruit fly, its short generation time, and ease of transformation. The *Drosophila* classic Gal4/UAS two-component expression system and its counterparts, LexA and Q*,* can be combined with one another and with site-specific recombination systems like Flp/FRT, Cre/LoxP or phiC31, yielding novel combinatorial systems for even tighter spatio-temporal gene expression control, clonal analysis, and lineage tracing [210]. The fruit fly genome is easily accessible using a broad range of genome engineering tools, including those based on classic transposition, site-specific recombinases and fosmid/BAC recombineering [211], as well as the emerging field of genome editing using designer nucleases [212]. Availability of an almost complete genomic sequence for over 12 species from genus *Drosophila* and dozens of various *D. melanogaster* strain genomes make fruit flies an excellent model for comparative genomics and population genetics. A large number of human disease-related genes that have homologs in the fruit fly [213,214] connected with powerful resources for QTL mapping and GWAS [89,90] make *Drosophila* an attractive model for studying the genetic basis of human disease. 

Rapid development of genome engineering techniques, especially those introducing synthetic approaches using designer DNA binding domains of TALEs [215,216] and the CRISPR system [217], will undeniably affect the *Drosophila* field in the next years. Completing the genome and transcriptome sequencing effort for additional fly species [218] will aid in further functional annotation of the *Drosophila* genome and fuel the evolutionary developmental biology field.
